# Constitutive Expression of *Arabidopsis* Senescence Associated Gene 101 in *Brachypodium distachyon* Enhances Resistance to *Puccinia brachypodii* and *Magnaporthe oryzae*

**DOI:** 10.3390/plants9101316

**Published:** 2020-10-06

**Authors:** Ning Wang, Na Song, Zejun Tang, Xiaojie Wang, Zhensheng Kang, Liangying Dai, Bing Wang

**Affiliations:** 1 Hunan Provincial Key Laboratory for Biology and Control of Plant Diseases and Insect Pests and College of Plant Protection, Hunan Agricultural University, Changsha 410128, Hunan, China; wangning@nwafu.edu.cn (N.W.); chinasong86@126.com (N.S.); Tangzj516@126.com (Z.T.); 2 State Key Laboratory of Crop Stress Biology for Arid Areas and College of Plant Protection, Northwest A&F University, Yangling 712100, Shaanxi, China; wangxiaojie@nwafu.edu.cn (X.W.); kangzs@nwsuaf.edu.cn (Z.K.)

**Keywords:** Senescence Associated Gene 101, salicylic acid, overexpression, *Puccinia brachypodii*, light microscopy

## Abstract

*Brachypodium distachyon*, as an effective model of cereal grains, is susceptible to most destructive cereal pathogens. *Senescence associated gene 101* (*SAG101*) has been studied extensively in *Arabidopsis*. *SAG101* is one of the important regulators of plant immunity. However, no homologous genes of *AtSAG101* were found in *B. distachyon*. In this study, the *AtSAG101* gene was transformed into *B. distachyon.* Three transgenic plant lines containing the *AtSAG101* gene were confirmed by PCR and GUS gene activity. There were fewer *Puccinia brachypodii* urediospores in the *AtSAG101*-overexpressing plants compared to wild type plants. *P. brachypodii* biomass was obviously decreased in *AtSAG101* transgenic plants. The length of infection hyphae and infection unit areas of *P. brachypodii* were significantly limited in transgenic plants. Moreover, there were small lesions in *AtSAG101* transgenic plants challenged by *Magnaporthe oryzae*. Salicylic acid accumulation was significantly increased, which led to elevated *pathogenesis-related* gene expression in transgenic *B. distachyon* inoculated by *P. brachypodii* or *M. oryzae* compared to wild type plants. These results were consistent with infected phenotypes. Overexpression of *AtSAG101* in *B. distachyon* caused resistance to *M. oryzae* and *P. brachypodii*. These results suggest that *AtSAG101* could regulate plant resistance in *B. distachyon*.

## 1. Introduction

Plants are continually threatened by a wide variety of potential pathogens in the environment. To cope with pathogens, plants have evolved multiple defense mechanisms to avoid or limit infection [1]. Plants produce an inducible hormone to prevent pathogen invasion and reproduction. Salicylic acid (SA) is a common signal that is essential for resisting pathogens [2]. Salicylic acid (SA) accumulation induces the basal defense or plant resistance (R) gene-pathogen avirulence gene interactions, which induce immunity when host tissues respond to fungi pathogen invasion. An important role of SA is to induce *pathogenesis-related* (*PR*) genes expression [3].

Senescence associated gene 101 (SAG101), a 3 lipase-like defense regulator, is an essential component of plant basal resistance against pathogenic strains [4]. SAG101 regulates the production of SA to limit pathogen growth. In Arabidopsis, SAG101 is required for plant resistance to *Pseudomonas syringae* pv. *tomato* strain DC3000 [5]. Moreover, SAG101 is necessary for R-mediated resistance to turnip crinkle virus [6]. Recently, it has been reported that SAG101a is required for *Xanthomonas campestris* pv. *vesicatoria* effector protein XopQ-induced resistance responses in *Nicotiana benthamiana* [7].

Cereal grains, such as rice and wheat, provide over 50% of dietary proteins and calories for people [8]. Cereal production is seriously limited and impacted by plant diseases. *Brachypodium distachyon* (*B. distachyon*) is an effective model for studying monocot species due to its small genome size, diploid inheritance, a short life-cycle, and simple growth requirements [9]. Due to its close evolutionary relationships to cereal grains, including wheat and rice, *B. distachyon* facilitates the research of cereal crop grass species in response to different environmental stresses, including of biotic stress [10].

*B. distachyon* is susceptible to many important cereal pathogens. *Magnaporthe oryzae* (rice blast) is the top fungal plant pathogen, which can cause devastating effects on rice [11]. The development and disease progression of *M. oryzae* (Guy11) fungus in *B. distachyon* and rice are similar [12]. *M. oryzae*-*B. distachyon* is the compatible interaction, which has emerged as a resource for studies on susceptible interactions [13]. Rust pathogens, namely, *Puccinia striiformis* f. sp. *tritici* (*Pst*, stripe rust), *P. graminis* f. sp. *tritici* (*Pgt*, responsible for wheat stem rust), and *P. triticina* (*Pt*, leaf rust), are third in the list of top 10 fungal plant pathogens, which are major disease threats to wheat production [11]. *Puccinia brachypodii* can infect many *Brachypodium* species, including *B. distachyon*. *P. brachypodii* is closely related to these three rust pathogens in wheat based on phylogenetic studies [14]. The compatible interaction between *B. distachyon* and *P. brachypodii* is being development as a model to study wheat and rust pathogen interactions.

*AtSAG101* is an important component of the plant basal resistance against pathogens in *Arabidopsis*. However, we did not identify homologous genes of *AtSAG101* in *B. distachyon.* In this study, we transformed *AtSAG101* into *B. distachyon* and showed that overexpression of *AtSAG101* in *B. distachyon* induced resistance to *P. brachypodii* and *M. oryzae*. These results inferred that *AtSAG101* confers resistance to pathogens in *B. distachyon*.

## 2. Results

### 2.1. Overexpression of AtSAG101 in B. distachyon

**Figure 1:**
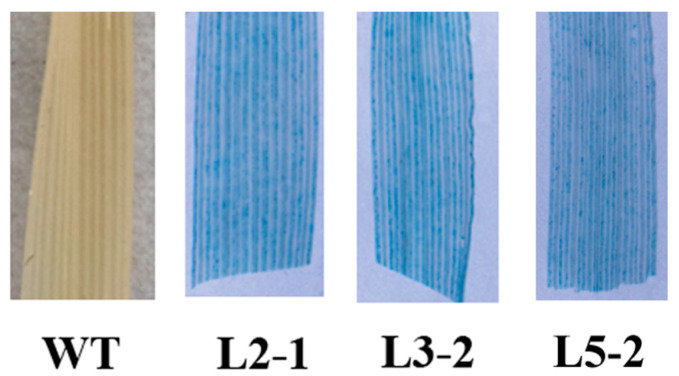
Confirmation of *Arabidopsis senescence associated gene 101* (*AtSAG101*) in transgenic *Brachypodium distachyon*. Histochemical staining of the β-glucuronidase (GUS) gene in *AtSAG101* transgenic *B. distachyon*. WT, wild type plants; L2-1, L3-2 and 5-2, transgenic *B. distachyon* plants.

There is considerable knowledge of the *AtSAG101* gene and its ability to enhance resistance to pathogens [5,7]. However, we did not find homologous genes of *AtSAG101* in *B. distachyon*. In this study, *AtSAG101* was transformed into *B. distachyon*. *AtSAG101* transgenic plants were identified by PCR. Three transgenic plant lines were successfully identified to contain the *AtSAG101* gene, named Lines 2-1, 3-2 and 5-2 (Figure S1). Furthermore, we analyzed *β-glucuronidase* (*GUS*) gene expression and activity in transgenic plants (Figure 1). Three transformed lines showed GUS activity, but wild type showed no detectable GUS phenotype using histological staining. These results demonstrated that *AtSAG101* was successfully transformed into *B. distachyon*.

### 2.2. AtSAG101 Transgenic Plants Induce Resistance to P. brachypodii 

**Figure 2:**
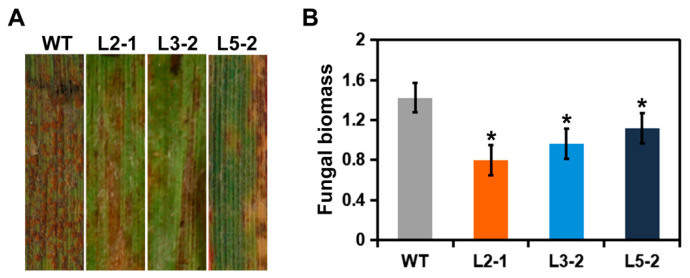
*AtSAG101* transgenic plants enhance resistance to *Puccinia brachypodii*. (**A**) Phenotypic changes in leaves of *AtSAG101* transgenic plants challenged with *P. brachypodii*. (**B**) Biomass of *P. brachypodii.* Significant differences were determined using Student’s *t*-test: *, *p* < 0.05. WT, wild type plants; L2-1, L3-2 and 5-2, transgenic *B. distachyon* plants.

To test the function of *AtSAG101* during *B*. *distachyon*-*P. brachypodii* interactions, *AtSAG101* transgenic plants were inoculated by the F-CO isolate of *P. brachypodii*. The *B*. *distachyon* Bd21-3 genotype is susceptible to *P. brachypodii*. Wild type (WT) plants produced numerous urediniospores 15 dpi after inoculation with F-CO. Compared to WT leaves, limited urediospore production was observed on leaves of *AtSAG101* transgenic plants (Figure 2A). *P. brachypodii* biomass was used to further verify phenotypes. *P. brachypodii* biomass was obviously decreased in the leaves of *AtSAG101* transgenic plants compared to WT plants (Figure 2B).

**Figure 3:**
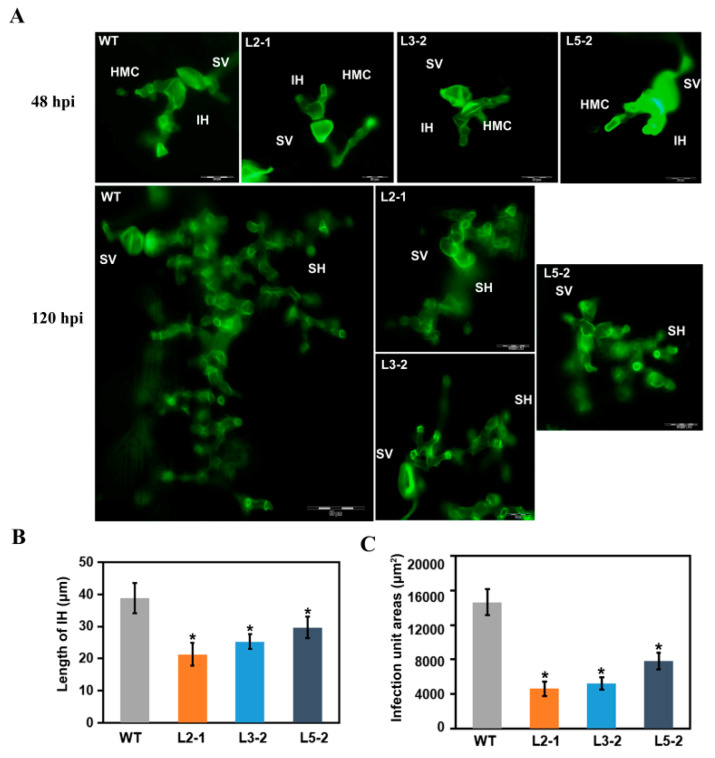
Histological determination of fungal growth in *AtSAG101* transgenic plants. (**A**) Histological observation of fungal development. (**B**) Length of infection hyphae was significantly reduced. (**C**) A significant decrease in infection unit areas. WT, wild type plants; L2-1, L3-2 and 5-2, transgenic *B. distachyon* plants; IH, infection hyphae; SH, secondary hyphae; HMC, haustorial mother cells; SV, substomatal vesicle (24 hpi, bar = 20 µm; 120 hpi, bar = 50 µm); *, *p* < 0.05.

Furthermore, histological changes in *AtSAG101* transgenic plants inoculated with F-CO were observed (Figure 3A). Length of infection hyphae (IH) and infection unit areas were observed and calculated. At 48 h after infection with the F-CO isolate, IH growth was significantly limited in *AtSAG101* transgenic plants (Figure 3B). Moreover, the *P. brachypodii* infection unit area was significantly reduced (*P* < 0.05) in *AtSAG101* transgenic *B. distachyon* leaves at 120 hpi compared to WT leaves (Figure 3C).

### 2.3. SA Levels Are Increased in AtSAG101-Overexpressing B. distachyon Leaves

**Figure 4:**
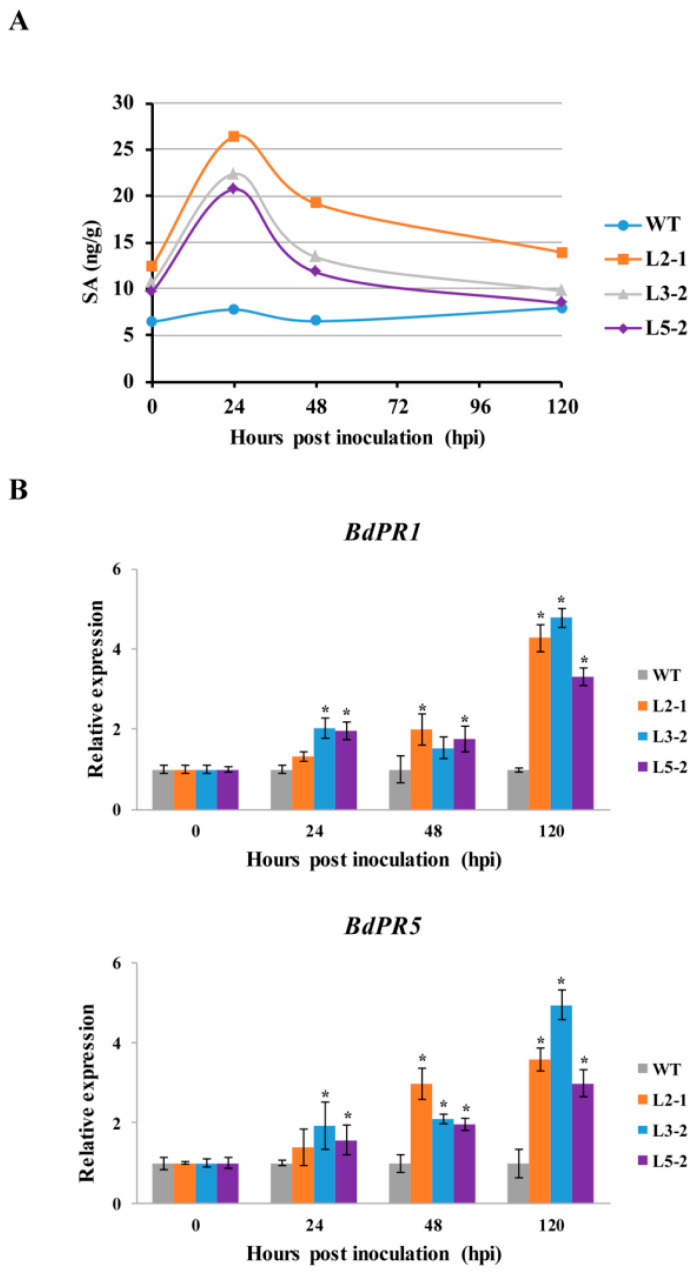
Detection of SA levels and defense-related genes in *AtSAG101* transgenic plants infected with *Puccinia brachypodii*. (**A**) SA accumulation in *AtSAG101* transgenic leaves. (**B**) Transcript levels of defense-related genes in *AtSAG101* transgenic leaves challenged with *P. brachypodii*. SA, salicylic acid; ng/mg, SA accumulation (ng) per fresh leaf weight (mg); WT, wild type plants; L2-1, L3-2 and 5-2, transgenic *B. distachyon* plants; *B. distachyon* (*Bd*) *PR1* gene (upper panel) and *BdPR5* gene (lower panel). *, *p* < 0.05.

SAG101 induces the production of SA to limit pathogen growth. To test a possible role of *AtSAG101* in regulating SA, we examined SA levels in *AtSAG101* transgenic *B. distachyon* leaves (Figure 4A). SA accumulation was slightly higher in transgenic *B. distachyon* leaves compared to WT plants when unchallenged by *P. brachypodii*. At 24 hpi, SA accumulation was obviously increased (the range from 20.69 to 26.39 ng/g) in transgenic plants infected with *P. brachypodii.* However, there was no obvious change in SA accumulation in WT plants challenged by *P. brachypodii*, indicating a different response to that shown earlier in WT plants challenged by *P. brachypodii*.

To determine if the expression levels of defense-related genes were affected in the *AtSAG101* transgenic plants after challenge by *P. brachypodii*, we selected *PR* genes for quantitative RT-PCR (qRT-PCR) analysis. The expression levels of *PR1*, and *PR5* were significantly increased in transgenic *B. distachyon* leaves infected with *P. brachypodii* at 24, 48, and 120 hpi (Figure 4B).

### 2.4. AtSAG101 Transgenic Plants Produce Resistance to Magnaporthe oryzae

**Figure 5:**
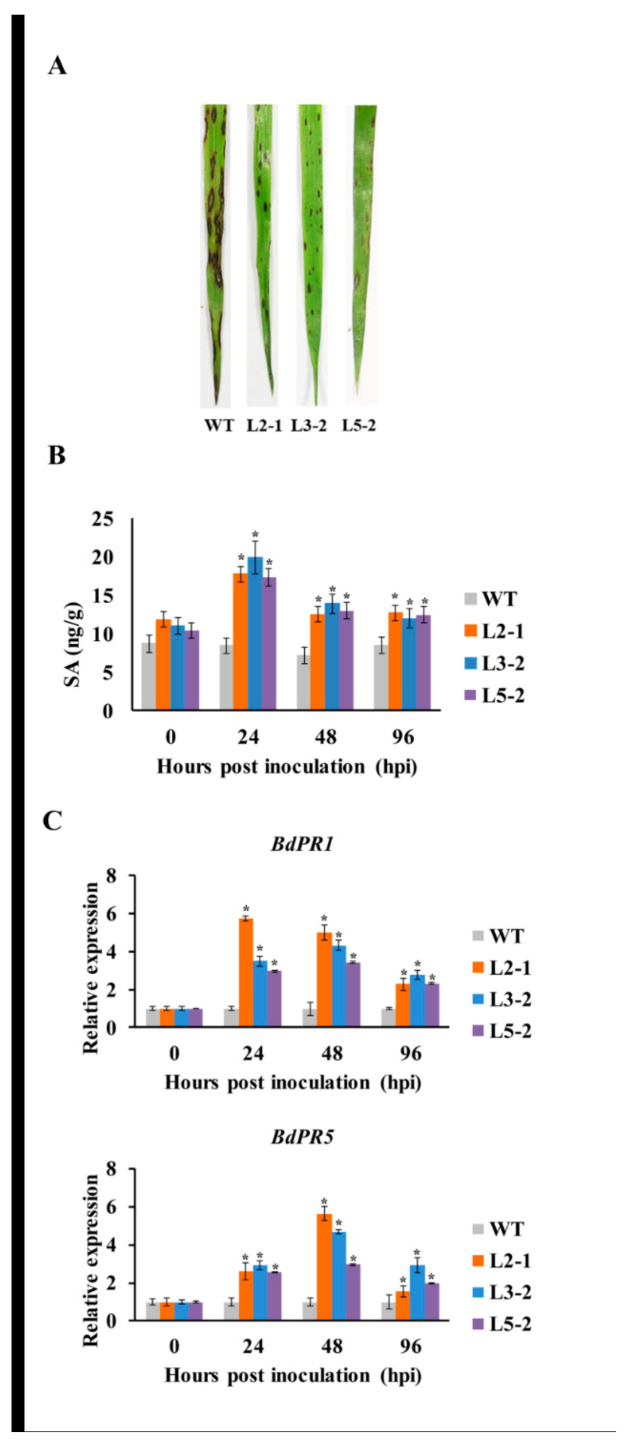
*AtSAG101* transgenic plants produce resistance to *Magnaporthe oryzae.* (**A**) Phenotypic changes in leaves of *AtSAG101* transgenic plants challenged with *M. oryzae.* (**B**) Salicylic acid (SA) accumulation in *AtSAG101* transgenic leaves infected with *M. oryzae*. (**C**) qRT-PCR detection of *PR* gene expression in *AtSAG101*-overexpressing *B. distachyon* leaves inoculated with *M. oryzae*. *PR*, *pathogenesis-related* gene; WT, wild type plants; L2-1, L3-2 and 5-2, transgenic *B. distachyon* plants; *B. distachyon* (*Bd*) *PR1* gene (upper panel) and *BdPR5* gene (lower panel). Significant differences were determined using Student’s *t*-test: *, *p* < 0.05.

*Magnaporthe oryzae* is an important rice fungus that can infect *B. distachyon.* In this study, we also inoculated *AtSAG101* transgenic plants with *M. oryzae* to test the function of *AtSAG101* during *B*. *distachyon*-*M. oryzae* interactions (Figure 5A). *AtSAG101* transgenic plants showed resistance to disease symptoms with small lesions, while large disease lesions were observed in WT plants at 5 dpi. Moreover, there was a 2.2-fold peak in SA accumulation in transgenic *B. distachyon* leaves challenged by *M. oryzae* at 24 hpi (Figure 5B). However, there was no obvious change in WT plants challenged with *M. oryzae*. The expression levels of *PR1* and *PR5* were significantly induced in transgenic plants challenged by *M. oryzae* at 24, 48, and 96 hpi (Figure 5C).

## 3. Discussion

Plants utilize multilayered defense strategies to limit or resist pathogen infection [1]. The lipase-like proteins, enhanced disease susceptibility1 (EDS1), SAG101 and phytoalexin deficient 4 (PAD4), are important regulators of plant immunity [15,16]. EDS1 and PAD4 are present in monocots and eudicots according to phylogenetic analysis, whereas SAG101 is not found in monocot genomes [17,18]. We also did not find homologous genes of *AtSAG101* in the *B. distachyon* genome, thereby inferring evolution of SAG101 in different plants. In this study, we transformed the *AtSAG101* gene into *B. distachyon* to determine if overexpression of the *AtSAG101* gene produces resistance to different pathogens. Three transgenic plant lines (Lines 2-1, 3-2 and 5-2) were successfully transformed plants containing the *AtSAG101* gene, which was confirmed by PCR and the GUS phenotype.

SAG101 is an essential component of plant basal resistance. In *Arabidopsis*, SAG101 plays important roles in resistance to bacterial pathogens and turnip crinkle virus [6]. In *N. benthamiana*, *NbSAG101a* is involved in resistance to *Xanthomonas campestris* pv. *Vesicatoria* [7]. *AtSAG101* induces resistance activity in *N. benthamiana*, inferring it may enhance resistance in other plants [19]. In this study, we demonstrated that overexpression of the *AtSAG101* gene produces resistance to *P. brachypodii* and *M. oryzae*. After inoculation with the *P. brachypodii* F-CO isolate, limited urediospore production was observed on *AtSAG101* transgenic plant leaves. The biomass and detailed histological analysis confirmed that IH and infection unit areas of *P. brachypodii* were inhibited in *AtSAG101* transgenic plants. These results were consistent with infected phenotypes. Moreover, *AtSAG101* transgenic plants showed resistance to disease symptoms with small disease lesions compared to WT plants during *B*. *distachyon*-*M. oryzae* interactions. These results indicated that *AtSAG101* may regulate immune signaling in different plants to multiply pathogenic strains.

SA, as an important plant defense signalling component, takes part in resistance to biotrophic pathogens. PAD4 is a key gene involved in pathogen-induced SA accumulation [20]. SAG101, which is thought to serve as a substitute for PAD4, functions in plant resistance [21]. There are different roles of PAD4 and SAG101 in regulating SA. Previously, we identified that SA accumulation is increased in *AtPAD4* transgenic *B. distachyon* resistance to *P. brachypodii* [22]. However, SA accumulation is not obviously changed in WT plants and *AtPAD4* transgenic plants lacking *P. brachypodii* infection. In this study, the SA levels were higher in *AtSAG101* transgenic plants compared to WT plants. Moreover, SA accumulation was obviously increased when transgenic plants were challenged with *P. brachypodii* and *M. oryzae*. These results suggested that *AtSAG101* transgenic plants enhance resistance to *P. brachypodii* and *M. oryzae* by increasing SA accumulation. We also found that PAD4 and SAG101 play different roles in mediating SA accumulation in *B. distachyon*. The resistance was not effectively activated in *B. distachyon* WT plants challenged with *P. brachypodii* and *M. oryzae*. SAG101 is not required for the SA-mediated induction of *R* genes [6]. We inferred overexpression of *AtSAG101* can directly activate SA pathway in *B. distachyon* to involve in plant immunity, which is independent on the SA-mediated induction of *R* genes. However, the complex mechanism of *AtSAG101* increasing SA accumulation needs to be studied further.

SA accumulation induces the expression of defense genes in response to virulent pathogens [23]. For example, the *TaMAPK4* (a wheat MAP kinase 4) gene is involved in regulating SA accumulation. In *TaMAPK4* knockdown plants, the expression levels of *TaPR1* and *TaPR5* are obviously reduced [24]. In this study, we found that the expression levels of *PR1* and *PR5* were significantly induced in *AtSAG101* transgenic plants challenged by *P. brachypodii* and *M. oryzae*. Therefore, these results suggested that the accumulation of PR proteins may lead to enhanced disease resistance in *AtSAG101*-overexpressing plants.

In summary, three transgenic plant lines were successfully transformed to contain the *AtSAG101* gene. We showed that overexpression of *AtSAG101* in *B. distachyon* enhances resistance to *M. oryzae* and *P. brachypodii*. SA accumulation was increased in *AtSAG101* transgenic plants challenged with *B. distachyon*, which lead to increased *PR* gene expression. Overexpression of *AtSAG101* can directly activate plant defense. The results suggested that broad-spectrum genes can be selected in the process of breeding. In particular, those downstream resistance factors which are directly involved in regulating the resistance pathway are valuable candidates in disease resistance breeding.

## 4. Materials and Methods

### 4.1. Plant and Fungal Materials

In this study, the *B*. *distachyon* Bd21-3 genotype, *P. brachypodii* F-CO isolate and *M. oryzae* RO1-1 isolate were used. Bd21-3 is susceptible to RO1-1 and F-CO. *B*. *distachyon* was grown in a 22 °C growth chamber with 16 h (h) of light and 8 h of dark. Three seedlings were grown 8 cm diameter pots with medium, and 5 weeks old plants were inoculated with F-CO. Each treatment contains WT and three different transgenic lines, three plants for each line. Parallel mock inoculations were performed using tap water. Inoculation and incubation of F-CO followed the procedures as previously described [22]. Inoculated leaves were harvested at 0, 24, 48 and 96 h post inoculation (hpi). The inoculation results were evaluated at 15 dpi. 

Five-week-old seedlings were challenged with *M. oryzae* spore (1 × 10^5^ spores mL^−1^) by spraying. Seedlings inoculated by RO1-1 were kept in a humid chamber at 28 °C. Disease symptoms were evaluated at 5 dpi. Three independent biological replications were performed for each treatment.

### 4.2. Gene Cloning and Agrobacterium Transformation

Total RNA of *A. thaliana* was extracted using the TrizolTM Reagent (Invitrogen, Carlsbad, CA) following the manufacturer’s instructions. To synthesize cDNA from RNA, a Revert Aid First-strand cDNA synthesis kit from Fermentas (www.thermosscientific.com/fermentas) was used. The *AtSAG101* gene (GenBank accession At5g14930, coding sequences without stop codon) was cloned from *Arabidopsis* leaves. A1611 bp PCR product was inserted into the pU1301 vector, which contains ubiquitin promotor and the reporter gene *β-glucuronidase* (*GUS*). The primers used for plasmid construction in the present study are listed in Supplementary Table S1. The pU1301- *AtSAG101* vector was constructed for the transgenic plants using the *Agrobacterium* AGL1. All constructs were verified by sequencing and double digestion, and they were then transformed into *B*. *distachyon* following the procedure of Vogel [25]. Three independent homozygous T3 lines with a high expression level of *AtSAG101* were selected for further study.

### 4.3. Histochemical Staining

Histochemical staining of leaves to detect *GUS* gene expression was conducted as previously described [24], except no chloramphenicol was used in the stain. Leaves were transferred into microtiter wells containing 500 μL of *GUS* staining solution (10 mM EDTA, 100 mM Na phosphate at pH 7, 1 mg/mL of X-Gluc, and 0.1% Triton X-100). Stained leaves were cleared in 95% ethanol to visualize localized staining. Sectioned samples were prepared for photography.

### 4.4. Endogenous SA Level Analysis

*B. distachyon* mutant and WT plant SA levels were analyzed by HPLC-MS [25]. The SA extraction was performed as previously described [22]. 250 mg of the frozen tissue was extracted and quantitated for SA. SA was extracted with MeOH-H_2_O-HOAc (90:9:1, *v*/*v*), the extract was evaporated and injected into liquid chromatography–electrospray ionization tandem mass spectrometry system (API 2000; AB SCIEX, United States of America (USA)). Standards of SA >99% (Fluka, Buchs, Switzerland) was made by diluting the standard solutions with the initial LC mobile phase (0.05% HOAc in H_2_O-MeCN, 85:15, *v*/*v*). SA quantitation was analyzed using the standard addition method of SA solutions (range of 50 to 1000 ng/mL). SA concentrations were calculated according to the detection results.

### 4.5. Histological Observation of Fungal Growth

*P. brachypodii* growth and development in mutant or wild type plants were characterized by histopathological analysis. Inoculated leaves were sampled at 48 h and 120 h, and leaves were treated and stained as previously described [25]. In brief, leaves were cut to 1.5 cm segments, which were fixed and decolorized in ethanol/trichloromethane (3:1 *v*/*v*) containing 0.15% (*w*/*v*) trichloroacetic acid for 3–5 days. For microscopic observation, leaves were treated and stained with WGA (wheat germ agglutinin conjugated to Alexa-488) (Invitrogen., Carlsbad, CA, USA). The lesion area and length were observed by an Olympus BX-53 microscope (Olympus Corp., Tokyo, Japan) and measured using CellScan Entry software. At least 30 randomly selected infection sites were measured, and 5 segments were randomly selected per treatment. Statistical analysis was performed using IBM SPSS 19 software (SPSS Inc., Chicago, IL, USA) with standard deviations and Tukey’s test.

### 4.6. PCR Analysis

qRT-PCR reactions of 25 μL included 25 ng cDNA, 10 μL Taq Mix and 0.5 μM of each primer. PCR conditions were as follows: 95 °C for 1 min, 30 times (95 °C for 20 s, 58 °C for 15 s, and 72 °C for 1 min), and 72 °C for 4min. To standardize the data, ubiquitin-conjugating enzyme 18 gene (UBC18) was the internal reference. qRT-PCR was performed using a 7500 Real-Time PCR System (Applied Biosystems). Electrophoresis was performed using a 1.5% agarose gel. qRT-PCR data were analyzed by the comparative 2^−ΔΔ*C*t^ method [26].

### 4.7. Biomass Analysis

To estimate changes in fungal biomass, the DNA level of *P. brachypodii EF1* was measured by quantitative PCR with the DNA level of reference gene *B. distachyon EF1*. Relative fungal growth was then calculated as a ratio (*Pb-EF1*/*Bd-EF1*) to reflect the amplification efficiency.

## Supplementary Materials

The following are available online at https://www.mdpi.com/2223-7747/9/10/1316/s1, Figure S1: PCR detection of *AtSAG101* in transgenic *Brachypodium distachyon*. The sequence was 2359 bp (ORF sequence 1578 bp + Vector sequence 781 bp). M, Molecular size marker; H, H_2_O blank control; PC, pU1301- *AtSAG101* vector positive control; WT, wild type plants; L2-1, L3-2 and L5-2: transgenic *B. distachyon* plants, Table S1: Primers for plasmid constructions and PCR.
