# Supplementary material for: Constitutive Expression of Arabidopsis Senescence Associated Gene 101 in Brachypodium distachyon Enhances Resistance to Puccinia brachypodii and Magnaporthe oryzae
Source: Plants (Basel). 2020 Oct 6;9(10):1316. doi: 10.3390/plants9101316 (PMC7650532; doi:10.3390/plants9101316)
Supplement: Supplementary file 1 [file plants-09-01316-s001.docx]

| **Primer** | | **Sequences (5'-3')** | **PCR fragment length (bp)** |
| --- | --- | --- | --- |
| OE-SAG101-F | GGGTACCCATGGAGTCTTCTTCTTCACTAAAAG | | 1627 |
| OE-SAG101-R | CGGATCCGTTGTGACTTACCATAACTCTCGTAC | |  |
| SAG101-ORF-F | ATGGAGTCTTCTTCTTCACTAAAAG | | 1611 |
| SAG101-ORF-R | TTGTGACTTACCATAACTCTCGTAC | |  |
| qRT-SAG101-F | GCGGTCACAGAGTTGCTAAA | | 123 |
| qRT-SAG101-R | ACATCTTCAGGGATCAATCTTG | |  |
| UBC18-F | GGAGGCACCTCAGGTCATTT | | 193 |
| UBC18-R | ATAGCGGTCATTGTCTTGCG | |  |
| pU1301-F | CTATCCTTCGCAAGACCCTTC | | 781+1611 |
| pU1301-R | CAATTCACACGTGATGGTGAT | |  |
| qRT-BdPR1-F | GACAGGAACCTGGAGGACTA | | 109 |
| qRT-BdPR1-R | AGTAGAGGTTCTCGCCGTAG | |  |
| qRT-BdPR5-F | GGGCAGAAAGTGATGAGGTATC | | 104 |
| qRT-BdPR5-R | GGTCTGGAACCATCACTTCTT | |  |

Table S1. Primers for plasmid constructions and PCR.


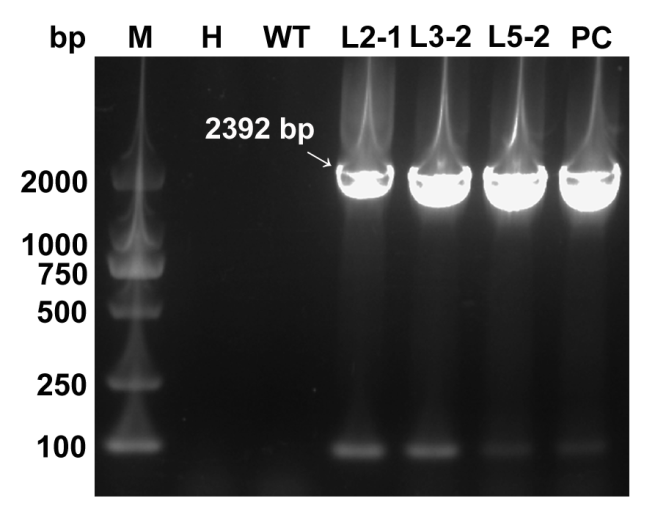


**Figure S1.** PCR detection of *AtSAG101* in transgenic *Brachypodium distachyon*. The sequence was 2392 bp (ORF sequence 1611bp + Vector sequence 781bp). M, Molecular size marker; H, H_2_O blank control; PC, pU1301- *AtSAG101* vector positive control; WT, wild type plants; L2-1, L3-2 and L5-2: transgenic *B. distachyon* plants.
